# Betaine alleviates hepatic lipid metabolism disorder in finishing pigs fed a low-energy diet through regulating m^6^A RNA methylation

**DOI:** 10.1186/s40104-025-01299-2

**Published:** 2025-12-02

**Authors:** Chan Liang, Runqi Fu, Daiwen Chen, Gang Tian, Jun He, Ping Zheng, Jie Yu, Junning Pu, Bing Yu

**Affiliations:** 1 Key Laboratory of Animal Disease-Resistance Nutrition, Ministry of Education, Ministry of Agriculture and Rural Affairs, Key Laboratory of Sichuan Province, Chengdu, Sichuan 611130 China; 2 Institute of Animal Nutrition, Sichuan Agricultural University, Chengdu, Sichuan 611130 China

**Keywords:** Betaine, Finishing pig, Lipid metabolism, Liver, Low energy diet, m^6^A

## Abstract

**Background:**

Low dietary energy levels can disrupt energy balance, causing metabolic disorders, particularly those involving in hepatic lipid metabolism. Betaine (BET), an important methyl donor, has demonstrated protective effects against liver diseases. However, its effects on hepatic lipid metabolism in pigs fed a low-net energy (NE) diet and the underlying mechanisms remain unclear. Thirty-two pigs (85.52 ± 2.27 kg) were randomly assigned to four treatments: N-NE group (normal NE diet, 2,475 kcal/kg NE), N-NEB group (normal NE diet + 1,500 mg/kg BET, 2,475 kcal/kg NE), R100-NE group (low-NE diet, 2,375 kcal/kg NE), and R100-NEB group (low-NE diet + 1,500 mg/kg BET, 2,375 kcal/kg NE). The experiment lasted 35 d.

**Results:**

There was no significant difference in growth performance among the groups (*P* > 0.05). Reducing dietary NE levels caused liver dysfunction and increased total glyceride concentration, accompanied by lipid metabolism disorders. BET supplementation in a low-NE diet exhibited hepatoprotective roles, as evidenced by increased TP concentration and reduced ALT level in serum (*P* < 0.05), as well as decreased fat content, adipocyte size, and total glyceride concentration in the liver (*P* < 0.05). Meanwhile, dietary BET alleviated low-NE diet-induced hepatic lipid metabolism disorder by downregulating mRNA expressions of genes related to fatty acid transport (*FABP3* and *CD36*) and lipogenesis (*SREBP1c* and *FASN*), while upregulating mRNA expressions involved in lipolysis (*CPT1* and *HSL*) (*P* < 0.05). Furthermore, dietary BET increased serum SAM concentration and the SAM/SAH ratio in pigs fed low-NE diets (*P* < 0.05), thereby providing sufficient methyl groups through regulating the activities of enzymes participated in BET metabolism. Mechanistically, BET increased m^6^A modification level and regulated mRNA and protein expressions of m^6^A modified proteins including METTL3, METTL14, WTAP, YTHDF1, and ALKBH5. Correlation analysis showed a significant association between m^6^A RNA methylation and hepatic lipid metabolism, suggesting that m^6^A RNA methylation may play a critical role in mediating hepatic lipid metabolism.

**Conclusions:**

Dietary BET supplementation in low-NE diets alleviated hepatic lipid metabolism disorders by regulating m^6^A RNA methylation, ultimately reducing hepatic lipid accumulation in finishing pigs.

**Graphical Abstract:**

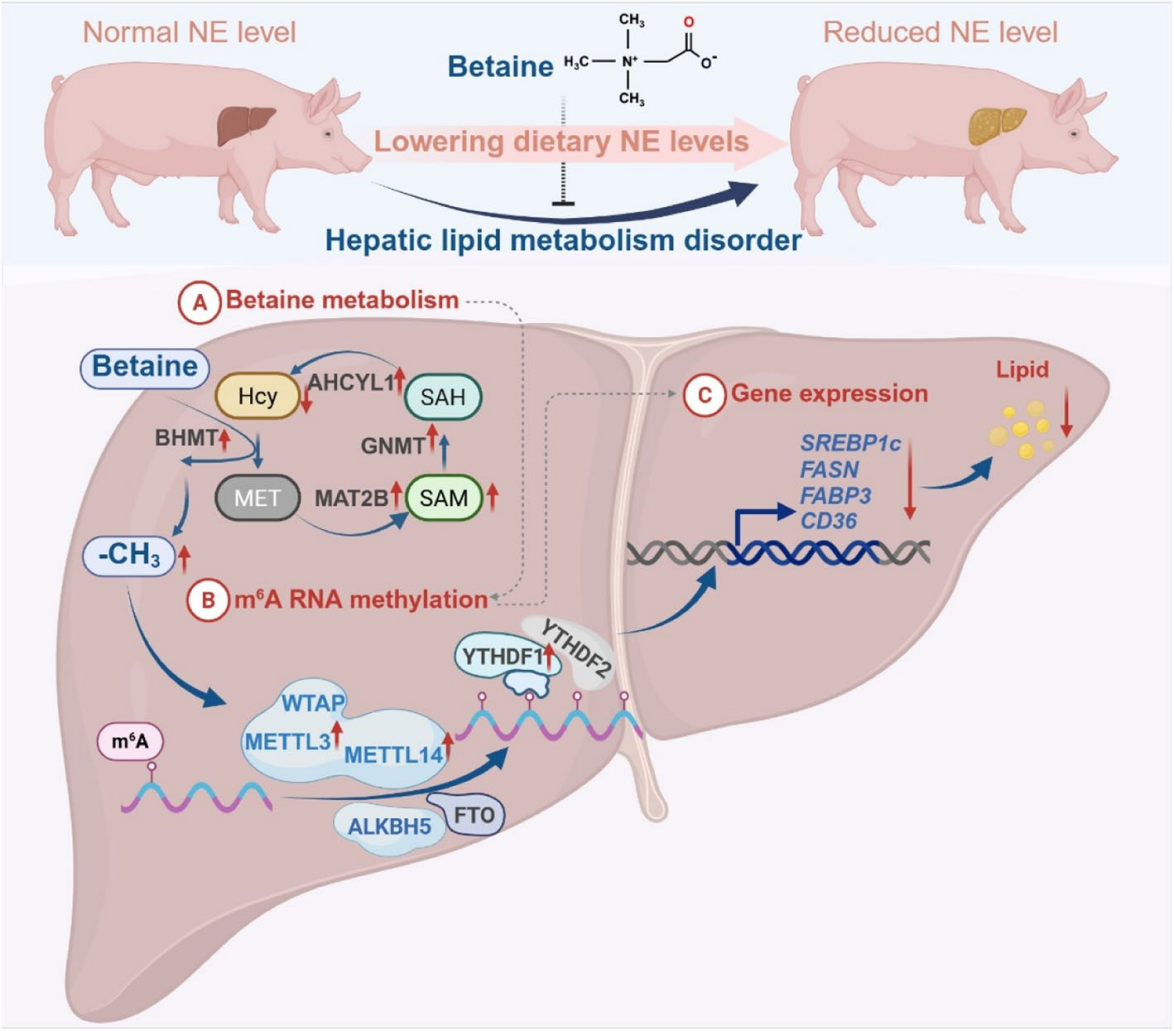

## Background

Dietary energy is a critical nutritional factor influencing growth and development, as well as a significant determinant of feed costs [1]. Given the current context of increasing global food insecurity, reducing dietary energy levels serves as a direct and effective approach to lower energy consumption and dietary costs [2]. However, this reduction introduces dual challenges. On one hand, animals may increase their feed intake to meet nutritional requirements, potentially negating cost-control measures [3]. On the other hand, reduced dietary energy often induces energy imbalance, resulting in metabolic disturbances and changes in body composition [4–6], particularly resulting in abnormalities in fat metabolism and storage [7, 8]. This subsequently diminishes livestock and poultry production performance and product quality. The liver, as the central organ regulating systemic metabolism in both animals and humans [9], is particularly affected by reduced dietary energy levels. It has been reported that nutritional deficiencies negatively impact liver health, evidenced by fat accumulation in the liver [10, 11]. Disorders in hepatic lipid metabolism not only hinder animal growth but also pose a significant risk for diseases such as fatty acid deficiency syndrome and fatty liver disease [12]. Therefore, it is vital to seek effective approaches to alleviate the detrimental effects of reduced dietary energy levels.

Betaine (BET), chemically known as *N*,*N*,*N*-trimethylglycine, is a compound that naturally occurs in various sources, including sugar beets, spinach, wheat, and shellfish [13]. BET has two primary biological roles, acting as an osmolyte and serving as a methyl donor [14]. Although BET is used as an osmotic protectant in tissues like the kidneys and brain, its primary role is as a methyl donor in liver metabolism [15]. As a feed additive for animals, BET has proven effective in promoting growth performance, regulating energy distribution, improving lipid metabolism, and supporting gut health [5, 16–19]. Previous studies have identified an interaction between BET and dietary energy, primarily examining the effects of BET on animal growth and development, as well as carcass characteristics [20–22]. Additionally, the hepatoprotective roles of BET have been well-documented in several liver diseases [23–26], though the underlying mechanisms are not yet fully understood. Notably, BET is essential for providing methyl groups through the production of S-adenosylmethionine (SAM), significantly influencing N^6^-methyladenosine (m^6^A) modification [27]. In our previous study, we observed that BET supplementation decreased flare fat content by regulating m^6^A RNA methylation in finishing pigs fed a low-energy diet [8]. Based on this, we hypothesize that BET might improve hepatic lipid metabolism through regulating m^6^A RNA methylation to alleviate the adverse effects in finishing pigs when fed low-energy diets. This study aimed to investigate the effects of BET in low-net energy (NE) diets on hepatic lipid metabolism and explore its underlying mechanisms in finishing pigs.

## Materials and methods

### Animals, diets and experimental design

Thirty-two finishing pigs [Duroc × (Landrace × Yorkshire)], initially weighing 85.52 ± 2.27 kg, were randomly assigned to four treatments (*n* = 8) using a 2 × 2 factorial design, corresponding to two dietary NE levels (2,475 or 2,375 kcal/kg) and two BET supplementation doses (0 or 1,500 mg/kg). Four treatments were the N-NE group (normal NE diet, 2,475 kcal/kg NE), N-NEB group (normal NE diet + BET, 2,475 kcal/kg NE), R100-NE group (low-NE diet, 2,375 kcal/kg NE), and R100-NEB group (low-NE diet + BET, 2,375 kcal/kg NE). The normal NE diets were based on NRC (2012) guidelines [28], whereas the low-energy diets were created by decreasing the NE level by 100 kcal/kg, maintaining the same crude protein and amino acid balance. The diet formula has been previously published [8]. Throughout the 35-d trial, all pigs were kept in a temperature‐controlled environment maintained at 18–22 °C and had unrestricted access to water and feed.

### Growth performance

All pigs after a 12-h fast were weighed individually on d 1 and 36 of the experiment, and daily feed intake for each pig was recorded. These data were used to calculate average daily gain (ADG), average daily feed intake (ADFI), and the ratio of feed to gain (F/G). In addition, net energy intake (NEI) was calculated from dietary NE content and ADFI, and the ratio of NEI to gain (NEI/G) was determined based on ADG and NEI.

$$\mathrm{ADG}\;(\mathrm g/\mathrm d)\:=\:(\mathrm{final}\;\mathrm{body}\;\mathrm{weight}\:-\:\mathrm{initial}\;\mathrm{body}\;\mathrm{weight})/\mathrm{number}\;\mathrm{of}\;\mathrm{trial}\;\mathrm{days};$$

$$\mathrm{ADFI}\;(\mathrm g/\mathrm d)\:=\:\mathrm{cumulative}\;\mathrm{feed}\;\mathrm{intake}\;\mathrm{of}\;\mathrm{each}\;\mathrm{pig}/\mathrm{number}\;\mathrm{of}\;\mathrm{trial}\;\mathrm{days};$$

$$\mathrm F/\mathrm G\:=\:\mathrm{ADFI}/\mathrm{ADG}.$$

### Sample collection and processing

On d 36 after weighing, 15-mL blood was collected from the anterior vena cava of each pig. The samples were first left undisturbed for 30 min and then centrifuged at 3,000 × *g* for 10 min to isolate serum for further analysis. Once blood was collected, the pigs were electrocuted and slaughtered. A midline incision was performed immediately post-slaughter to obtain tissue samples. Initially, after removing the surface fascia and wiping dry the moisture, the liver, kidneys, heart, and spleen were weighed and recorded to calculate organ indices. Subsequently, middle portions of the left lateral lobe of the liver were collected and stored at −80 °C until analysis. In addition, a 1-cm^3^ sample from the medial lobe of the liver was fixed in a 4% paraformaldehyde solution for later analysis.

### Serum indicator analysis

The concentrations of total protein (TP), albumin (ALB), blood urea nitrogen (BUN) and glucose (GLU), and the activities of alkaline phosphatase (AKP), aspartate aminotransferase (AST) and alanine aminotransferase (ALT) in serum were measured using the commercial kits (Nanjing Jiancheng Bioengineering Institute, Nanjing, China). The contents of SAM and S-adenosylhomocysteine (SAH) in serum were employed by an ultra-performance liquid chromatography (UPLC). The standards for SAM and SAH were purchased from Cayman Chemical (No. 13956, USA) and Sigma-Aldrich (No. A9384, USA), respectively. Serum homocysteine (Hcy) content was determined by the corresponding swine ELISA kit (Jiangsu Meimian Biotechnology Co., Ltd., Yancheng, China).

### Liver fat and histological analysis

The content of crude fat in liver was measured following the AOAC method 920.39 [29]. Before the crude fat measurement, liver samples underwent pre-processing to eliminate moisture, as described by Fu et al. [30]. The liver tissues, fixed in a 4% paraformaldehyde solution, were taken out and subsequently processed into slices through dehydration, embedding, and sectioning. These slices were dewaxed and stained with hematoxylin and eosin (H&E) for histological analysis. The stained tissues were observed using an Olympus BX43 microscope (Olympus Corp., Tokyo, Japan) at magnifications of 200 × and 400 ×.

### Quantitative real-time (qRT)-PCR

Total RNA extraction, reverse transcription, and PCR procedure were adopted from the methods described by Fu et al. [8]. Briefly, total RNA from the liver sample was extracted using the RNAiso Plus (No. 9109, TaKaRa, Dalian, China). The integrity of RNA was analyzed by agarose gel electrophoresis, and subsequently the concentration and purity of RNA were assessed with a NanoDrop ND-2000 spectrophotometer (Thermo Scientific, USA). For each sample, 1 μg of RNA was subjected to reverse transcription to synthesize cDNA using the PrimeScript™ FAST RT reagent kit (No. RR092A, TaKaRa, Dalian, China). The qRT-PCR was conducted using the TB Green^®^ Premix Ex Taq™ II FAST qPCR kit (No. CN830A, TaKaRa, Dalian, China) with the QuantStudio™ 5 Flex System (Thermo Scientific, USA). The primer sequences for β-actin and all target genes were presented in Table 1. Each target gene’s relative expression was calculated using the 2^−ΔΔCt^ method.

**Table 1:** Gene primer sequences utilized for real-time PCR

Gene	Nucleotide sequence of primers (5'→3')		Product length, bp	Accession No.
*FABP3*	F	TTGTGACACTGGATGGAGGC	25	NM_001099931.1
	R	AGTTTGCCTCCATCCAGT		
*CD36*	F	CTGTGGACTCATTGCTGGTGCTG	179	XM_021102279.1
	R	AAAACTGTCTGTAAACTTCCGTGCCT		
*SCD*	F	AATGCCACCTGGCTGGTAAA	195	XM_021072070.1
	R	TTCCACTGGTTCAGAGGGGA		
*SREBP1c*	F	GCGACGGTGCCTCTGGTAGT	218	XM_021066226.1
	R	CGCAAGACGGCGGATTTA		
*FASN*	F	GCCGAGTACAGCGTCAACAACC	173	NM_001099930.1
	R	TGGTCCTTCTTCATCAGCGGGAT		
*ACC*	F	TGTCCACTCAAGCATACCTCCCA	136	XM_021066238.1
	R	GCTACCATGCCAATCTCATTTCCTCC		
*CPT-1*	F	AGTCATGGTGGGCGACTAACTATGTG	169	XM_021091195.1
	R	ATCATGGCGTGGACAGCGTTC		
*HSL*	F	ACCTGACACTGCATGACCTG	164	NM_214315.3
	R	GGTGCTAATCTCGTCTCGGG		
*PGC-1α*	F	GGACTGACATCGAGTGTGCT	126	NM_213963.2
	R	TGAGTCCACCCAGAAAGCTG		
*LPL*	F	AACGTCATTGTGGTGGACTGGCT	165	XM_021072174.1
	R	TCCAAGGCTGTATCCCAGGAGGTG		
*BGT1*	F	TAAGGAGCCGAGTCCCTTCT	188	XM_005664091.3
	R	CTGTTGCTCCCAGCTCAGAAT		
*BHMT*	F	GGGAAGTGCTTTGGACATGC	107	NM_001200042.1
	R	TGGAAGGGTTGTATGGCCTG		
*MAT2B*	F	GCTCTCTATCCACTTTGTTCCC	116	NM_001142832.1
	R	TGTACACAGCTCTGCCAAGA		
*GNMT*	F	GCTTTCGACAAGTGGGTCATT	187	NM_001110419.2
	R	ACCGCACCATGCTTGTGAT		
*AHCYL1*	F	GCAGCAAACCAACTCCAAGG	134	XM_021088652.1
	R	CCTGAGCACGTTTCCTGAGT		
*METTL3*	F	ACACTGCTTGGTTGGTGTCA	151	XM_003128580.5
	R	AATCTTTCGAGTGCCAGGGG		
*METTL14*	F	TGTGTTTACGCAAGTGGGGT	179	XM_003129231.6
	R	AATGAAGTCCCCGTCTGTGC		
*WTAP*	F	AGAATCTGCACGCAGGGAAA	111	XM_021101851.1
	R	CTAGGCTGCTGGACTTGCTT		
*YTHDF1*	F	CCCCAGAGAACGAAAGGACA	157	XM_021078235.1
	R	AGTAGCTGGACAAGTAGGGGT		
*YTHDF2*	F	AACAAGGGTCCTGTGGCAAA	148	XM_005665152.3
	R	GCTGTGTCTGTTGCCCTACT		
*FTO*	F	GCATGGCTGCTTATTTCGGG	154	NM_001112692.1
	R	TGCATCAGAGCCCTTCACTG		
*ALKBH5*	F	TACTTCTTCGGCGAGGGCTA	191	XM_021067995.1
	R	TGGTAGTCGTTGATGACGGC		
*β-actin*	F	TGGAACGGTGAAGGTGACAGC	177	XM_003124280.5
	R	GCTTTTGGGAAGGCAGGGACT		

### Western blot

Hepatic protein was extracted using RIPA lysis buffer (No. P0013B, Beyotime, Shanghai, China) with PMSF protease inhibitor buffer (No. ST2573, Beyotime, Shanghai, China). The protein concentration of each sample was quantified using the BCA protein assay kit (Thermo Scientific, USA) and subsequently adjusted to a same concentration. Using SDS-PAGE electrophoresis, the denatured protein samples of equal concentrations (25 µg) were separated (80 V for 15 min followed by 180 V for 40 min) and transferred (100 V for 90 min) onto 0.45-μm PVDF membranes. The membranes were blocked with 5% nonfat milk for 1 h and incubated overnight at 4 °C with primary antibodies (Table 2). Then, the membranes were incubated for 1 h with goat anti-rabbit/mouse IgG-HRP secondary antibody (Beyotime, Shanghai, China). The protein bands were visualized through chemiluminescent detection using the BeyoECL Moon reagent (No. P0018FM, Beyotime, Shanghai, China) and ChemiDoc™ XRS Imager System (Bio-Rad, CA, USA), and analyzed quantitatively with Image Lab software (version 5.1, Bio-Rad, CA, USA). The β-actin was employed as a housekeeping control for protein loading standardization.

**Table 2:** The primary antibody information of proteins

Protein	Dilution rate	Product number	Manufacturer
β-actin	1:1,000	#3700	Cell Signaling Technology
BGT1	1:1,000	67700-1-Ig	Proteintech
BHMT	1:2,000	15965-1-AP	Proteintech
MAT2B	1:1,000	15952-1-AP	Proteintech
GNMT	1:1,000	18790-1-AP	Proteintech
AHCYL1	1:1,000	10658-3-AP	Proteintech
METTL3	1:1,000	ab195352	Abcam
METTL14	1:1,000	26158-1-AP	Proteintech
WTAP	1:5,000	60188-1-Ig	Proteintech
YTHDF1	1:1,000	66745-1-Ig	Proteintech
YTHDF2	1:1,000	24744-1-AP	Proteintech
FTO	1:1,000	sc-271713	Santa Cruz Biotechnology
ALKBH5	1:2,000	67811-1-Ig	Proteintech
m^6^A	1:1,000	#56593	Cell Signaling Technology

### Dot blot

A dot blot analysis was performed to assess the m^6^A methylation level in the liver tissue [31]. Briefly, total RNA was extracted from the liver sample using the RNAiso Plus (No. 9109, TaKaRa, Dalian, China), and its concentration and purity were determined. The RNA was denatured by heating at 95 °C for 3 min, then rapidly cooled on ice. Subsequently, the RNA solution was carefully placed onto a nylon membrane (Beyotime, Shanghai, China) to achieve RNA concentrations of 250, 500, and 1,000 ng/spot, respectively. Following a 10-min ultraviolet irradiation to crosslink the RNA, it was blocked for 1 h using 5% nonfat milk, and incubated overnight at 4 °C with m^6^A antibody (Table 2). Subsequently, the membrane was incubated for 1 h with anti-rabbit IgG-HRP secondary antibody (Beyotime, Shanghai, China). Finally, the membrane was stained with 0.02% methylene blue to verify equal RNA loading, with the m^6^A levels subsequently normalized accordingly. The signals were visualized using a ChemiDoc™ XRS Imager System (Bio-Rad, CA, USA), and analyzed with Image Lab software (version 5.1, Bio-Rad, CA, USA).

### Statistical analysis

The results were subjected to statistical analysis utilizing the MIXED procedure of SAS software (Version 9.4, SAS Institute Inc., NC, USA) through a two-way ANOVA, followed by Tukey’s multiple comparison method. The statistical model employed was *y*_*ijk*_ = *µ* + *α*_*i*_ + *β*_*j*_ + *α*_*i*_ × *β*_*j*_ + *e*_*ijk*_, where *y*_*ijk*_ represents an observation, *μ* represents the overall mean, *α*_*i*_ represents the fixed effect of dietary NE level (*i* = 2,475 or 2,375 kcal/kg), *β*_*j*_ represents the fixed effect of dietary BET level (*j* = 0 or 1,500 mg/kg), *α*_*i*_ × *β*_*j*_ is the interaction between NE and BET, and *e*_*ijk*_ is the random error. Data were presented as means with the standard error of the mean (SEM). Significance was determined by *P* < 0.05, and 0.05 ≤ *P* < 0.10 was considered as a trend. Pearson’s correlation analysis was used to analyze the correlation between lipid metabolism and methylation-related genes in the liver.

## Results

### Growth performance, NEI and NEI/G

As shown in Table 3, no significant differences were observed in growth performance, NEI, and NEI/G among the groups (*P* > 0.05). However, compared to the N-NE group, the R100-NE group demonstrated a slight enhancement in ADFI, F/G, and NEI/G by 3.98%, 6.29%, and 2.01%, respectively (*P* > 0.05). Furthermore, in comparison to the R100-NE group, the R100-NEB group decreased ADFI, F/G, NEI, and NEI/G by 9.35%, 5.61%, 9.40%, and 5.64%, respectively (*P* > 0.05).

**Table 3:** Effects of BET supplementation in low-NE diets on growth performance, NEI and NEI/G in finishing pigs

Items	2,475 kcal/kg		2,375 kcal/kg		SEM	*P*-value		
	N-NE	N-NEB	R100-NE	R100-NEB		NE	BET	NE × BET
Initial BW, kg	85.61	85.50	85.46	85.51	0.86	0.77	0.90	0.73
Final BW, kg	127.05	127.10	126.10	124.44	1.88	0.15	0.51	0.48
ADFI, g/d	3,573.89	3,565.26	3,716.05	3,368.76	151.79	0.80	0.10	0.12
ADG, g/d	1,183.93	1,188.57	1,161.07	1,112.14	45.23	0.17	0.53	0.45
F/G	3.02	3.01	3.21	3.03	0.08	0.21	0.25	0.31
NEI, Mcal/d	8.85	8.82	8.83	8.00	0.37	0.11	0.11	0.13
NEI/G, Mcal/kg	7.47	7.45	7.62	7.19	0.20	0.79	0.26	0.32

*BW* Body weight, *ADFI* Average daily feed intake, *ADG* Average daily gain, *F/G* Feed‐to‐gain ratio, *NEI* Net energy intake, *NEI/G* Net energy intake-to-gain ratio

### Serum biochemical parameters

In Table 4, reducing dietary NE levels decreased TP concentration and increased BUN and ALT levels in serum (*P* < 0.05). Conversely, BET supplementation in a low-NE diet significantly increased TP concentration and reduced BUN and ALT levels in the serum of finishing pigs (*P* < 0.05).

**Table 4:** Effects of BET supplementation in low-NE diets on serum biochemical parameters in finishing pigs

Items	2,475 kcal/kg		2,375 kcal/kg		SEM	*P*-value		
	N-NE	N-NEB	R100-NE	R100-NEB		NE	BET	NE × BET
TP, µg/mL	59.54^a^	58.42^ab^	53.81^b^	62.39^a^	2.24	0.046	0.62	0.01
ALB, g/L	265.26	246.00	247.23	246.31	6.75	0.20	0.15	0.18
BUN, mmol/L	4.40^b^	4.30^b^	5.52^a^	4.31^b^	0.23	0.02	0.01	0.02
GLU, mmol/L	4.60	4.54	4.13	4.31	0.12	< 0.01	0.57	0.27
AKP, U/L	6.44	6.80	7.13	6.46	0.50	0.73	0.75	0.31
AST, U/L	6.34	7.08	7.65	7.00	0.48	0.15	0.92	0.11
ALT, U/L	20.57^b^	20.26^b^	24.44^a^	19.95^b^	1.68	0.16	0.06	0.10

*TP* Total protein, *ALB* Albumin, *BUN* Urea nitrogen, *GLU* Glucose, *AKP* Alkaline phosphatase, *AST* Aspartate aminotransferase, *ALT* Alanine aminotransferase

^a,^^b^Means with different superscript letters in a row were significantly different (*P* < 0.05)

### Organ indices

**Fig. 1:**
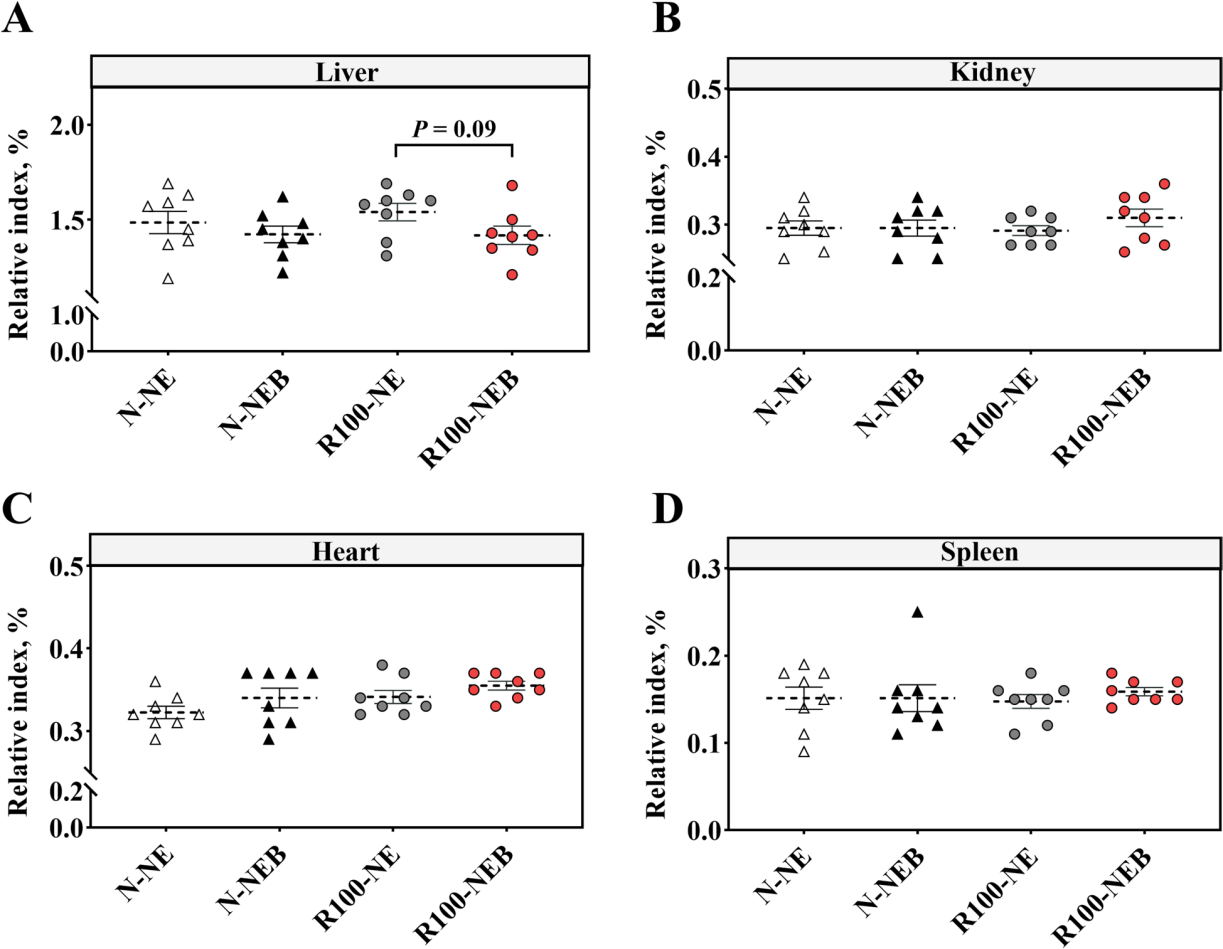
Effects of BET supplementation in low-NE diets on organ indices in finishing pigs. **A** Liver index. **B** Kidney index. **C** Heart index. **D** Spleen index. Data are shown as mean ± SEM

As presented in Fig. 1, there was no significant difference in organ indices among the groups (*P* > 0.05), however, dietary BET supplementation in a low-NE diet showed a decreasing trend in liver index (Fig. 1A, *P* = 0.09).

### Liver fat deposition and lipid metabolism

**Fig. 2:**
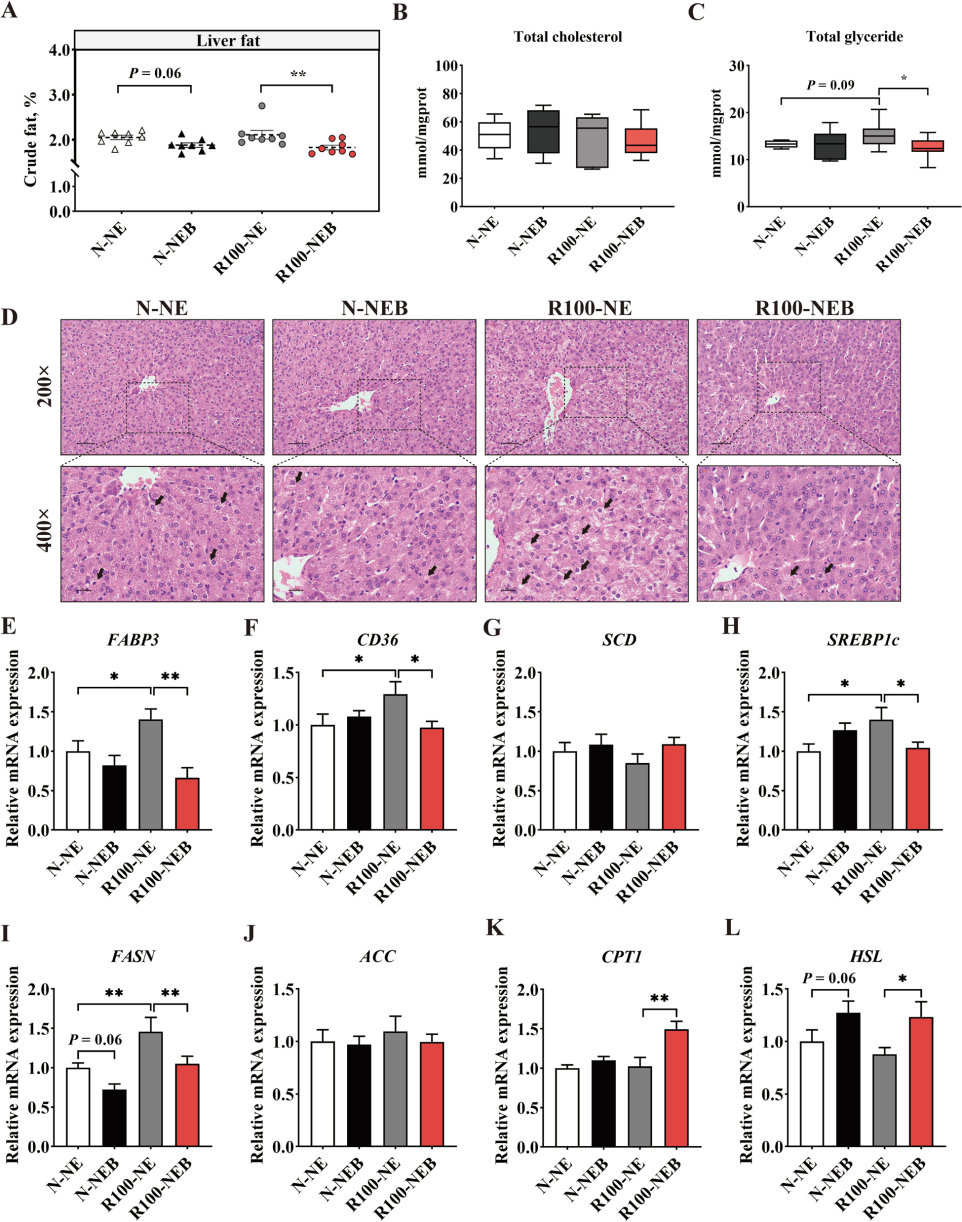
Dietary BET supplementation in low-NE diets attenuated the deposition of liver fat through regulating lipid metabolism in finishing pigs. **A** Fat content in the liver. **B** Total glyceride concentration in the liver. **C** Total cholesterol concentration in the liver. **D** Hematoxylin and eosin (H&E) staining section of adipocyte distribution at 200 × and 400 × magnification.** E**−**L** Lipid metabolism-related gene mRNA expression in the liver. Data are shown as mean ± SEM. ^*^P < 0.05; ^**^P < 0.01

As shown in Fig. 2, compared with the N-NE group, the N-NEB group tended to decrease the content of liver fat (Fig. 2A, *P* = 0.06) and reduce the size of liver adipocytes (Fig. 2D). Moreover, reducing dietary NE levels tended to increase total glyceride concentration (Fig. 2C, *P* = 0.09) while obviously increase the size of adipocytes in the liver (Fig. 2D). However, BET supplementation in a low-NE diet significantly reversed these changes, as evidenced by the decreased liver fat content (Fig. 2A, *P *< 0.01), reduced total glyceride concentration (Fig. 2C, *P* < 0.05), and smaller adipocyte size (Fig. 2D).

Compared to the N-NE group, pigs in the N-NEB group exhibited a trend of up-regulation of fatty acid synthetase (FASN) mRNA expression (Fig. 2I, *P* = 0.06) and down-regulation of hormone-sensitive lipase (*HSL*) mRNA expression (Fig. 2L, *P* = 0.06). Under low-NE condition, genes related to fatty acid transport and lipogenesis showed significant upregulation, including fatty acid-binding protein 3 (*FABP3*), cluster of differentiation 36 (*CD36*), sterol regulatory element-binding protein-1c (*SREBP1c*), and *FASN* (Fig. 2E−I, *P* < 0.05). However, adding BET to the low-NE diet not only significantly reversed the mRNA expressions of these genes (Fig. 2E−I,* P* < 0.05) but also significantly enhanced carnitine palmitoyl transferase 1 (*CPT1*) and *HSL* mRNA expressions (Fig. 2K and L, *P* < 0.05).

### Serum SAM, SAH and Hcy concentrations

**Fig. 3:**
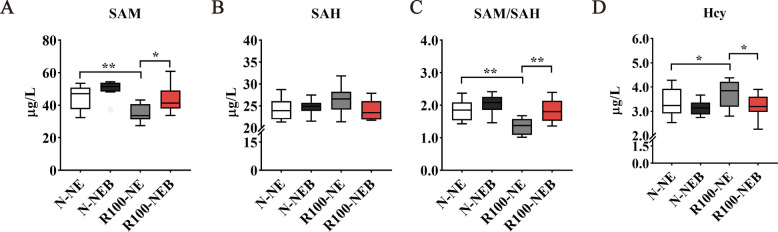
Dietary BET supplementation in low-NE diets regulated the serum SAM, SAH, and Hcy concentrations in finishing pigs. **A** Serum SAM concentration. **B** Serum SAH concentration. **C** Serum SAM/SAH ratio. **D** Serum Hcy concentration. Data are shown as mean ± SEM. ^*^P < 0.05; ^**^P < 0.01

BET is a highly active methyl donor crucial for sustaining normal lipid metabolism in the body. To evaluate whether the methylation ability of BET is affected by dietary NE levels, the BET metabolism in serum were analyzed (Fig. 3). Compared to the N-NE group, the R100-NE group markedly reduced serum SAM concentration and SAM/SAH ratio (Fig. 3A and C, *P* < 0.01) and increased serum Hcy concentration (Fig. 3D, *P* < 0.05). However, BET supplementation significantly alleviated the reductions in serum SAM concentration and SAM/SAH ratio, as well as the elevation of serum Hcy induced by a low-NE diet (Fig. 3A, C and D, *P* < 0.05).

### Hepatic BET metabolism

**Fig. 4:**
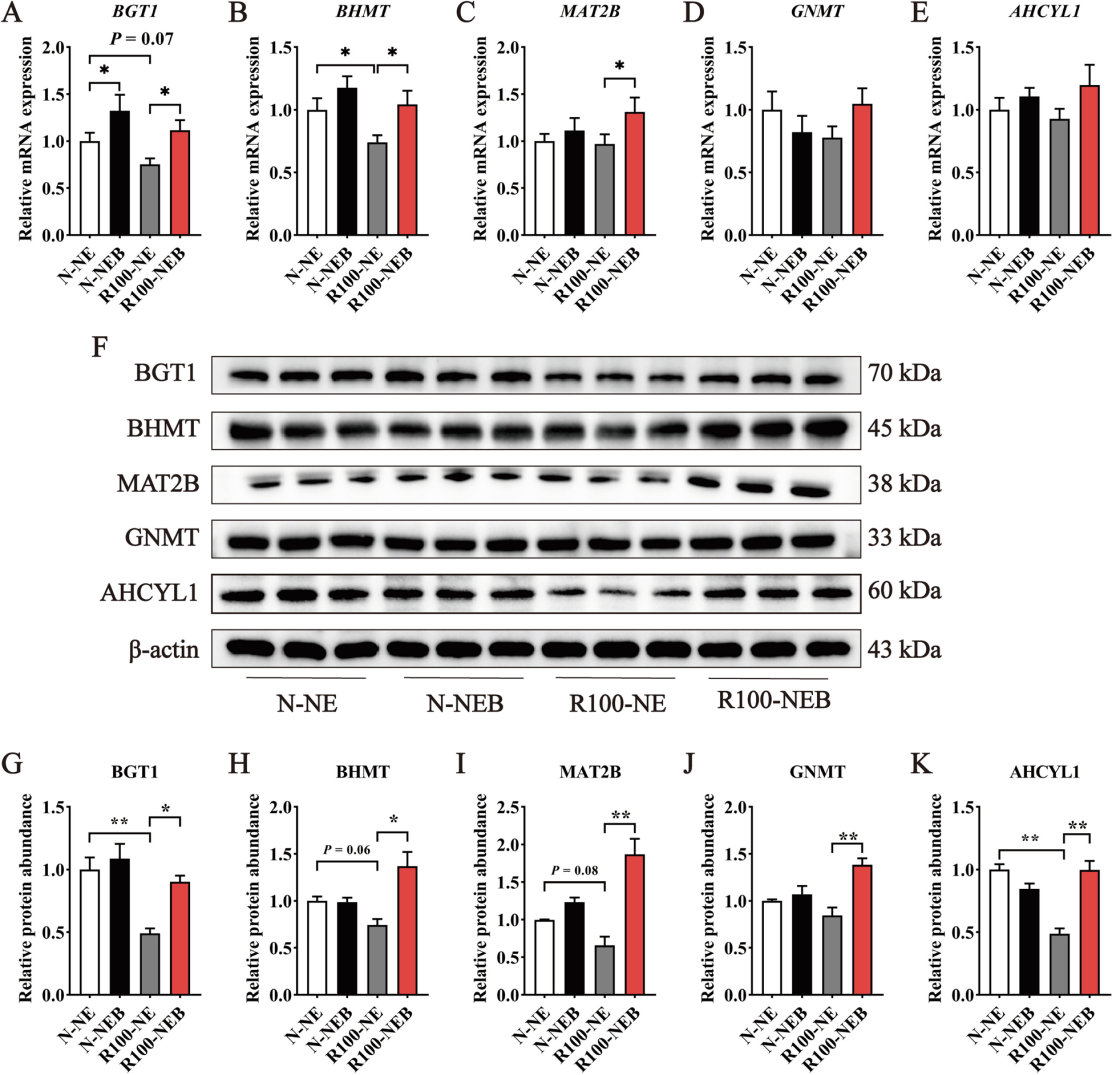
Dietary BET supplementation in low-NE diets ameliorated hepatic BET metabolism in finishing pigs. **A**−**E** BET metabolism-related gene mRNA expression in the liver. **F**−**K** BET metabolism-related protein expression in the liver. Data are shown as mean ± SEM. ^*^P < 0.05; ^**^P < 0.01

The changes in serum metabolites of BET are associated with its metabolic processes in the liver; therefore, further analysis of the critical genes involved in hepatic BET metabolism is warranted. As shown in Fig. 4, reducing dietary NE levels decreased the mRNA expressions of betaine/γ-aminobutyric acid transporter 1 (*BGT1*) (Fig. 4A, *P* = 0.07) and betaine-homocysteine methyltransferase (*BHMT*) (Fig. 4B, *P* < 0.05), with corresponding decreases in protein expressions of BGT1 (Fig. 4F and G, *P* < 0.01), BHMT (Fig. 4F and H, *P* = 0.06), methionine adenosyl transferase 2B (MAT2B) (Fig. 4F and I, *P* = 0.08), and adenosylhomocysteine hydrolase-like protein 1 (AHCYL1) (Fig. 4F and K, *P* < 0.01) in the liver of pigs. Nevertheless, BET supplementation in the low-NE diet not only upregulated mRNA expressions of *BGT1*, *BHMT*, and *MAT2B* (Fig. 4A−C,* P* < 0.05), but also increased protein expressions of BGT1, BHMT, MAT2B, glycine-N-methyltransferase (GNMT), and AHCYL1 (Fig. 4F−K,* P* < 0.05).

### Hepatic m^6^A methylation

**Fig. 5:**
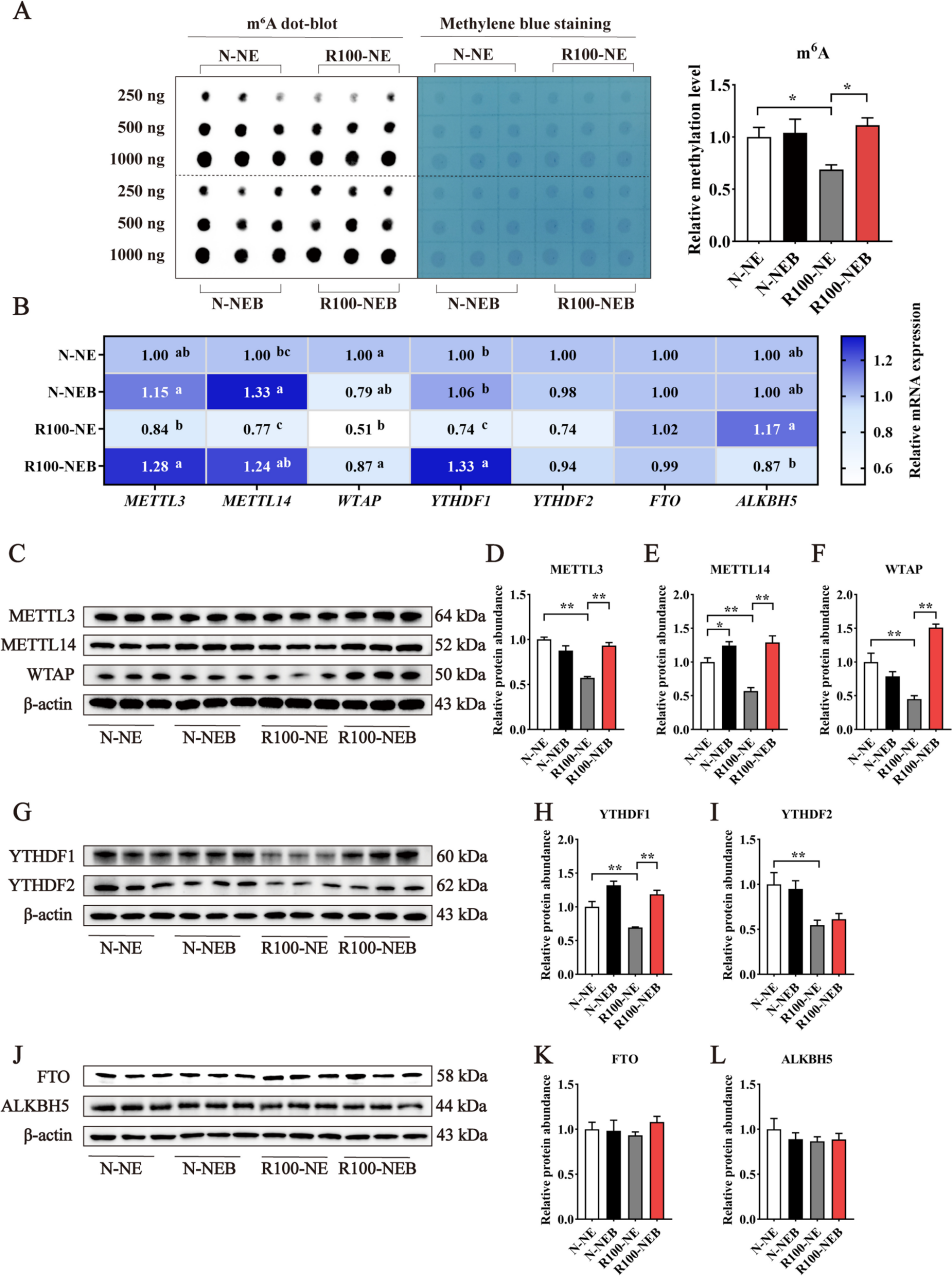
Dietary BET supplementation in low-NE diets ameliorated hepatic m^6^A methylation in finishing pigs. **A** Total m^6^A methylation level in the liver. **B** The heatmap of mRNA abundances of m^6^A methylation related genes in the liver. **C**−**L** Protein expressions of m^6^A methylation related genes in the liver. Data are shown as mean ± SEM. ^*^P < 0.05; ^**^P < 0.01

Betaine is primarily known for its role in methyl donation, which contributes to various biological function. Consequently, we analyzed the level of m^6^A methylation in the liver using a method of dot blot. Results showed that total m^6^A methylation level in the liver was decreased by lowering dietary NE levels, however, BET supplementation effectively restored the m^6^A methylation level in a low-NE diet (Fig. 5A, *P* < 0.05). Moreover, the R100-NE group decreased the mRNA expressions of key genes involved in DNA methylation, including Wilms tumor 1 associated protein (*WTAP*) and YTH domain family protein 1 (*YTHDF1*) (Fig. 5B, *P* < 0.05), and also decreased the protein expressions of methyltransferase-like 3 (METTL3), METTL14, WTAP, YTHDF1, and YTHDF2 (Fig. 5C−I,* P* < 0.01). In contrast, the mRNA and protein levels of demethylases such as AlkB homolog 5 (ALKBH5) and fat mass and obesity-associated protein (FTO) remained unaffected by dietary NE reduction (Fig. 5J−L,* P* > 0.05). Nevertheless, BET supplementation not only increased mRNA and protein expressions of METTL14 in the N-NE diet (Fig. 5B and E, *P* < 0.05), but also obviously elevated mRNA and protein expressions of METTL3, METTL14, WTAP, and YTHDF1 and decreased *ALKBH5* mRNA level in a R100-NE diet (Fig. 5B and I, *P* < 0.01).

### Correlation analysis of lipid metabolism and m^6^A RNA methylation-related genes in the liver

**Fig. 6:**
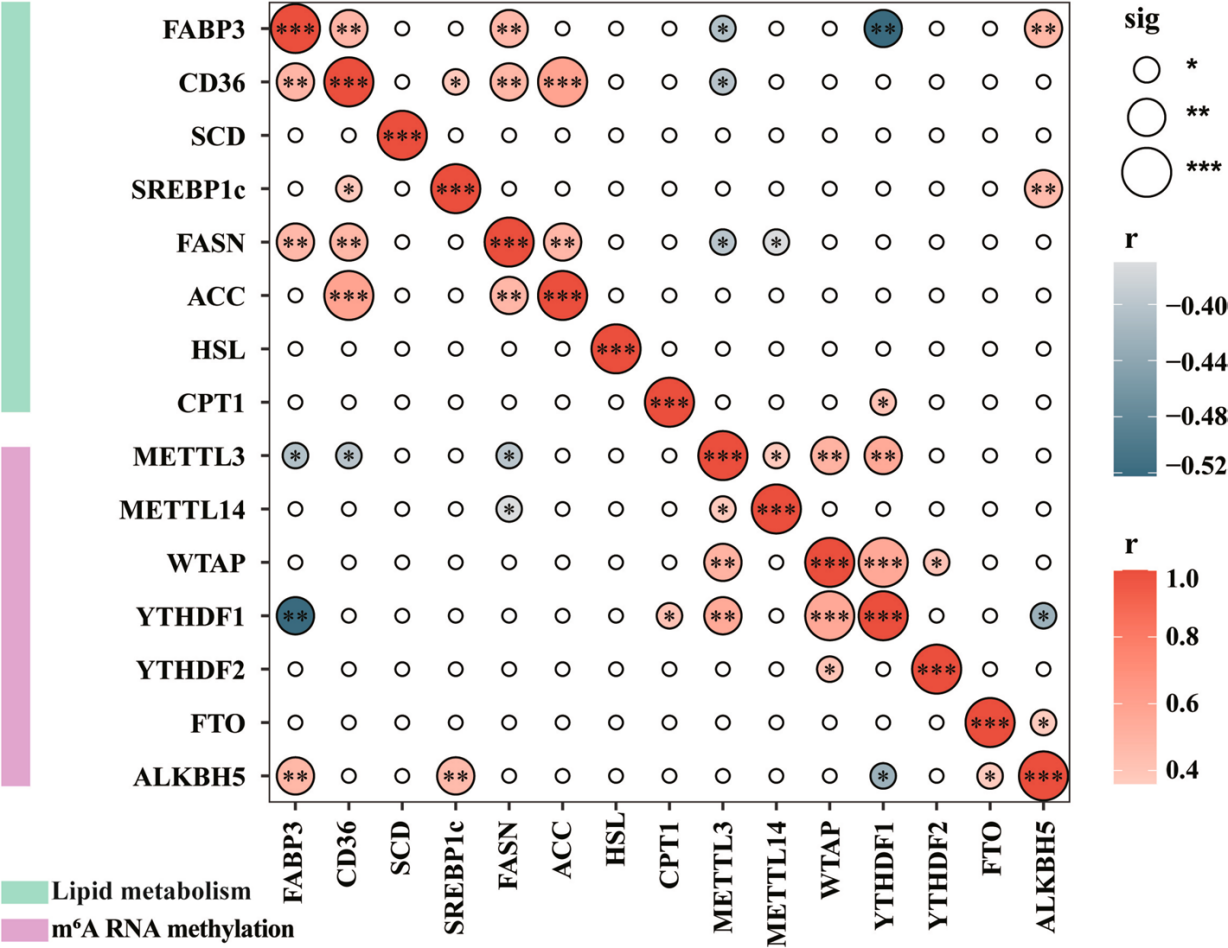
Correlation analysis of lipid metabolism and m^6^A RNA methylation-related genes in the liver. Red and blue colors represent positive and negative correlations, respectively. ^*^P < 0.05; ^**^P < 0.01; ^***^P < 0.0001

As shown in Fig. 6, *METTL3* exhibited significant negative correlations with *FABP3*, *CD36*, and *FASN*. Similarly, *METTL14* also exhibited apparent negative correlations with *FASN*. Moreover, *YTHDF1* was negatively correlated with *FABP3*, while *ALKBH5* was positively correlated with *FABP3* and *SREBP1c*.

## Discussion

Since energy costs constitute the largest proportion of animal feed expenses, reducing dietary energy levels represents a direct and effective approach to lowering feed costs [1]. Interestingly, studies have demonstrated that decreasing dietary energy density prompts animals to compensate by increasing their voluntary feed intake [32–34]. In the present study, when dietary NE level was reduced by 100 kcal/kg, pigs exhibited a corresponding increase in ADFI of 3.98%, reflecting an adaptive mechanism to satisfy their energy requirements. However, this adjustment was accompanied by a concurrent rise in both F/G and NEI/G, indicating that unrestricted access to low-NE diets may not be conducive to cost-effective production. An earlier study has highlighted the potential of BET supplementation to influence energy partitioning in growing pigs [35]. Consistent with this, our findings revealed that BET supplementation in R100-NE diet resulted in a reduction of ADFI and NEI, without adversely affecting ADG. In addition, F/G and NEI/G were reduced in finishing pigs, mirroring observations from our previous studies conducted on growing pigs [5]. These results collectively demonstrated that BET supplementation can effectively reduce feed intake while improving energy conversion efficiency, thereby offering a viable strategy to mitigate the adverse effects of low-NE diets and enhance overall cost control in swine production systems.

Investigating the intricate relationship between dietary BET and energy metabolism inevitably involves studying how BET regulates energy utilization in the body [36]. It is widely agreed that insufficient energy intake can disrupt metabolic homeostasis, leading to a series of metabolic health problems [37]. This effect is particularly pronounced in lipid metabolism. For instance, previous studies have shown that rats fed a low-energy diet exhibited ectopic fat deposition [7]. Similar results have been observed in the study where low-energy diets were used to intervene in the growth of fattening pigs [8]. In this study, pigs fed the low-NE diet exhibited a reduced TP level and an increased BUN level, which mirrored metabolic disorders. However, these abnormal changes were reversed by dietary BET supplementation, which probably indicated that BET might improve energy utilization efficiency and compensate for energy deficiencies. Liver plays an essential role in whole-body metabolism in both animals and humans [38], and its health is usually assessed using serum AST and ALT [39]. In this study, reducing dietary NE level increased serum ALT level, suggesting that a low-NE diet induced liver dysfunction [5]. Previous studies have demonstrated that BET supplementation can protect liver health and even reverse the progression of liver dysfunction [16, 23, 40, 41]. Consistently, we found a significant reduction in serum ALT level when pigs were fed a low-NE diet supplemented with BET. These findings collectively suggested that adding BET to low NE diets could improve metabolic function and protect liver health in pigs.

Organ index serves as a key indicator for organ growth, metabolism, and functional status [42]. In our study, pigs fed a low-NE diet with BET exhibited a decreasing trend in liver index, while no significant changes were observed in other organ indices. These data further support the conclusion that the liver is an indispensable site for BET-mediated biological processes [43]. Moreover, the liver serves as a critical role in regulating lipid metabolism. Loss of hepatic metabolic flexibility can lead to triglycerides accumulation as lipid droplets, eventually resulting in metabolic disturbances [44]. In this study, pigs fed a low-NE diet exhibited elevated hepatic total glyceride concentrations and enlarged adipocyte size. This observation may be attributed to the imbalanced dietary energy-to-protein ratio in the low-NE diets, which disrupted hepatic lipid metabolism. Previous studies have demonstrated that BET supplementation can protect against chronic liver diseases [45, 46]. In our study, addition of BET alleviated low-NE diets-induced hepatic lipid metabolic disorders, as evidenced by reductions in hepatic fat content, total glyceride concentration, and adipocyte droplet size. Dysregulation of hepatic lipid metabolism leads to ectopic fat accumulation, a process involving fatty acid transport, lipogenesis, and lipolysis [47], which are regulated by several critical molecules. Under low-NE condition, the mRNA expressions of genes involved in fatty acid transport, including *FABP3* and *CD36*, were significantly increased, contributing to fat uptake. Additionally, the mRNA levels of several key lipid synthesis genes such as *SREBP1c* and *FASN* were also elevated after administration of low-NE diets. However, BET supplementation in low-NE diets significantly downregulated the mRNA expressions of these genes, suggesting that BET may reduce fatty acid transport and suppress lipogenesis, thereby attenuating hepatic lipid accumulation. In addition, we observed that BET supplementation in a low-NE diet increased the mRNA levels of *CPT1* and *HSL*, which were key regulators of lipolysis. Li et al. [48] showed that CPT1 and HSL were negatively regulated by FASN, and their overexpression promoted the reduction of adipose tissue content. These findings suggested that BET might attenuate low-NE diet-induced hepatic lipid metabolism disorders by suppressing lipogenesis and enhancing lipolysis. Overall, our data indicated that dietary BET supplementation could mitigate the adverse effects of low-NE diets on liver health and metabolic function in pigs.

As a highly active methyl donor, the protective roles of BET are primarily associated with its modulation of genomic methylation [49]. BET has been demonstrated to be a critical methyl donor required for the syntheses of SAM [14]. SAM is converted to SAH after donating its methyl group, which is subsequently hydrolyzed to produce Hcy and adenosine [50]. Notably, SAM/SAH serves as a critical indicator of DNA methylation capacity. An elevation in the SAM/SAH ratio induces global DNA hypermethylation [51]. Studies have been reported BET promoted SAM generation, increased the SAM/SAH ratio, and reduced Hcy accumulation in rats and geese [51, 52]. Similarly, we also observed dietary BET significantly alleviated the reductions in serum SAM concentration and the SAM/SAH ratio, as well as the elevation in serum Hcy level induced by low-NE diets, thus suggesting that a low-NE diet with BET can ensure an adequate supply of methyl groups for pigs.

Since BET-dependent methylation predominantly occurs in the liver, we further measured the parameters associated with BET metabolism in hepatic tissues. BGT1, an important transporter, facilitates hepatic BET uptake into hepatocytes to enable its participation in the methionine cycle [53]. BHMT is the core enzyme in betaine metabolism, catalyzing the transfer of methyl groups from BET to Hcy, which directly determines the methyl-donating capacity of BET [54, 55]. Previous research has proved that BHMT activity significantly increased in the presence of sufficient dietary BET [56]. In this study, we observed that administration of BET reversed the decrease in mRNA and protein expressions of BGT1 and BHMT caused by low-NE diets. This suggested that adding BET into low-NE diets enhanced hepatic BET uptake and then activated BET-related metabolic responses. The balance between SAM and SAH is critical for maintaining cellular methylation capacity, and any disruption to this balance can impair cellular function [57]. Key enzymes involved in the synthesis and degradation of SAM and SAH include methionine adenosyltransferase (MAT), GNMT, and adenosylhomocysteinase (AHCY) [58–60], which collectively regulate methyl donor homeostasis. In our study, BET with NE-diets led to an upregulation of MAT2B, GNMT, and AHCYL1 protein expressions, which was consistent with the observed changes in serum SAM/SAH ratio. Thus, BET supplementation to a low-NE diet provided sufficient methyl groups to protect liver health in finishing pigs through regulating enzymes activities involved in BET metabolism.

N^6^-methyladenosine is an epigenetic RNA modification that involved in multiple biological processes, including mRNA splicing, export, translation, and degradation [61]. Increasing evidence demonstrates the critical role of m^6^A methylation in adipogenesis [62, 63]. For example, a previous study has shown that mRNA m^6^A methylation suppresses adipogenesis in porcine adipocytes [64]. Interestingly, BET has been demonstrated to increase mRNA m^6^A level in a dose-dependent manner and exhibit a negative relationship with adipogenesis [64]. We found that BET increased total m^6^A methylation level while decreasing fat content in the liver of pigs fed a low NE diet, which aligns with our previous results on flare fat [8]. These findings suggest that BET decreases the accumulation of hepatic fat under low-NE dietary conditions, potentially mediated by enhanced m^6^A methylation level. m^6^A methylation is a dynamic and reversible process regulated by three classes of proteins: methyltransferases (writers, e.g., METTL3, METTL14, and WTAP), m^6^A recognition-binding proteins (readers, e.g., YTHDF family), and demethylases (erasers, e.g., FTO, ALKBH5) [65]. The m^6^A “writers” catalyze methyl group transfer from SAM to RNA. Conversely, the “eraser” enzymes are responsible for reversing RNA methylation, dynamically regulating the balance of m^6^A modifications in response to cellular cues [66]. The “reader” proteins recognize m^6^A sites, regulating the biological function of m^6^A RNA modifications [67]. Our results showed that BET supplementation significantly increased mRNA and protein levels of METTL3, METTL14, WTAP, and YTHDF1 while reducing *ALKBH5* mRNA expression in a R100-NE diet. This suggested that BET addition in low-NE diets could enhance the methylation reaction by upregulating m^6^A writers and downregulating erasers. Previous studies have demonstrated a direct link between m^6^A RNA methylation and lipid metabolism regulation, with METTL3 further shown to be positively correlated with m^6^A levels and negatively correlated with adipogenesis [64]. Our study also found an inverse relationship between *METTL3* and liver lipogenic genes, including *FABP3*, *CD36*, and *FASN*. Similarly, *METTL14* exhibited a negative correlation with *FASN*, while *YTHDF1* was negatively correlated with *FABP3*. Additionally, *ALKBH5*, an m^6^A “eraser”, positively correlated with lipogenesis-related genes including *FABP3* and *SREBP1c* in the liver. Furthermore, studies have shown that overexpression of an m^6^A “eraser” (FTO) promotes excessive lipid accumulation in the liver via the SREBP1c pathway [68]. Collectively, these findings further confirm that BET alleviates hepatic lipid metabolism disorders in a low-NE diet via enhancing m^6^A modification process.

## Conclusion

Dietary BET supplementation in low-NE diets effectively alleviated hepatic lipid metabolism disorders in finishing pigs by regulating m^6^A methylation, ultimately reducing hepatic lipid accumulation. These findings highlight the critical role of BET in promoting hepatic health and provide a reasonable strategy for the application of low-NE diets in swine production systems.

## Data Availability

The datasets used during the current study are available from the corresponding author upon reasonable request.

## Abbreviations

ACC: Acetyl-CoA carboxylase
ADFI: Average daily feed intake
ADG: Average daily gain
AHCYL1: Adenosylhomocysteine hydrolase-like protein 1
AKP: Alkaline phosphatase
ALB: Albumin
ALKBH5: AlkB homolog 5
ALT: Alanine aminotransferase
AST: Aspartate aminotransferase
BET: Betaine
BGT1: Betaine/γ-aminobutyric acid transporter 1
BHMT: Betaine-homocysteine methyltransferase
BUN: Blood urea nitrogen
BW: Body weight
CD36: Cluster of differentiation 36
CPT1: Carnitine palmitoyl transferase 1
F/G: The ratio of feed to gain
FABP3: Fatty acid-binding protein 3
FASN: Fatty acid synthetase
FTO: Fat mass and obesity-associated protein
GLU: Glucose
GNMT: Glycine-N-methyltransferase
Hcy: Homocysteine
HSL: Hormone-sensitive lipase
m^6^A: N^6^-methyladenosine
MAT2B: Methionine adenosyl transferase 2B
METTL14: Methyltransferase-like 14
METTL3: Methyltransferase-like 3
NE: Net energy
NEI: Net energy intake
NEI/G: The ratio of net energy intake to gain
SAH: S-Adenosylhomocysteine
SAM: S-Adenosylmethionine
SAM/SAH: The ratio of S-adenosylmethionine to S-adenosylhomocysteine
SCD: Stearoyl-CoA desaturase
SREBP1c: Sterol regulatory element-binding protein-1c
TP: Total protein
WTAP: Wilms tumor 1 associated protein
YTHDF1: YTH domain family protein 1
YTHDF2: YTH domain family protein 2
